# Experimental analysis of bladder cancer-associated mutations in *EP300* identifies EP300-R1627W as a driver mutation

**DOI:** 10.1186/s10020-023-00608-7

**Published:** 2023-01-16

**Authors:** Mayao Luo, Yifan Zhang, Zhuofan Xu, Shidong Lv, Qiang Wei, Qiang Dang

**Affiliations:** Department of Urology, Nanfang Hospital, Southern Medical University, Guangzhou, 510515 Guangdong China

**Keywords:** EP300, Bladder cancer, Missense mutation, Driver mutation

## Abstract

**Background:**

Bladder cancer (BCa) is the most common malignant tumor of the urinary system, with transitional cell carcinoma (TCC) being the predominant type. *EP300* encodes a lysine acetyltransferase that regulates a large subset of genes by acetylating histones and non-histone proteins. We previously identified several bladder cancer-associated mutations in EP300 using high-throughput sequencing; however, the functional consequences of these mutations remain unclear.

**Methods:**

Bladder cancer cells T24 and TCC-SUP were infected with shEP300 lentiviruses to generate stable EP300 knockdown cell lines. The expression levels of EP300, p16 and p21 were detected by real-time PCR and western blots. The transcriptional activity of p16 and p21 were detected by dual luciferase assay. Cell proliferation assay, flow cytometric analyses of cell cycle, invasion assay and xenograft tumor model were used to measure the effect of EP300-R1627W mutation in bladder cancer. Immunoprecipitation was used to explore the relationship between EP300-R1627W mutation and p53. Structural analysis was used to detect the structure of EP300-R1627W protein compared to EP300-wt protein.

**Results:**

we screened the mutations of EP300 and found that the EP300-R1627W mutation significantly impairs EP300 transactivation activity. Notably, we demonstrated that the R1627W mutation impairs EP300 acetyltransferase activity, potentially by interfering with substrate binding. Finally, we show that EP300-R1627W is more aggressive in growth and invasion in vitro and in vivo compared to cells expressing EP300-wt. We also found that the EP300-R1627W mutation occurs frequently in seven different types of cancers.

**Conclusion:**

In summary, our work defines a driver role of EP300-R1627W in bladder cancer development and progression.

**Supplementary Information:**

The online version contains supplementary material available at 10.1186/s10020-023-00608-7.

## Introduction

Bladder cancer (BCa) is the most common malignant tumor of the urinary system, with transitional cell carcinoma (TCC) being the predominant type (Ferlay et al. 2018; Siegel et al. 2021). It is estimated that approximately 340,000 new cases and 170,000 deaths occur worldwide every year (Patel et al. 2020). To provide a global landscape of genetic lesions in TCC BCa, we carried out a high-throughput sequencing of 97 clinical cases with TCC BCa and identified massive genetic variants, especially in chromatin remodeling genes (Gui et al. 2011). Genetic mutations that are positively selected and contribute to the development and progression of cancers are considered drivers (Colaprico et al. 2020). Although modern high-throughput sequencing unveiled many cancer-associated genetic lesions, only a few of them are likely to be biologically meaningful(Kahn 2011). Therefore, the functional assessment of these lesions is necessary to identify drivers in order to translate genomic information into biological knowledge and clinical applications. Particularly in terms of the BCa-associated genetic lesions identified by our previous high-throughput sequencing study, the functional consequences of these mutations are still unclear.

EP300, or p300, was originally identified through its interaction with the adenoviral-transforming protein E1A (Stein et al. 1990). It is a 300-kilodalton protein encoded by the *EP300* gene. EP300 belongs to the KAT3 family of lysine acetyltransferases, which contain a histone acetyltransferase (HAT) domain and is capable of acetylating histone and non-histone proteins (Black et al. 2006; Das et al. 2009). EP300 is known as a global transcriptional coactivator for a number of transcription factors and plays a critical role in tumorigenesis pathways(Iyer et al. 2004). It regulates transcription by epigenetically acetylating chromatin and/or transcription factors or by serving as scaffolds that bridge sequence-specific DNA binding factors and the basal transcriptional machinery (Chan and La Thangue 2001). Indeed, it was demonstrated to transactivate the transcription of p21^Waf1/Cip1^, a tumor suppressor, and control the transcriptional regulation of p16^Ink4A^ (Wang et al. 2008; Wong et al. 2010; Xiao et al. 2000). EP300 has also been reported to function as a cancer suppressor by interacting with and acetylating TP53 and BRCA-1 (Grossman 2001; Mullan et al. 2006). According to the cBioPortal database, the EP300 mutation rate in bladder cancer is 16.6%, ranking first in pan-cancer analysis (Cerami et al. 2012). In bladder cancer, patients with EP300 mutations were associated with a positive prognosis and higher tumor mutational burden (TMB) scores, which was reported to be a biomarker for immunotherapy (Zhu et al. 2020). In addition, inhibition of CBP and p300 impaired bladder cancer cell proliferation and induced apoptosis by decreasing c-Myc expression (Li et al. 2019). However, the function of EP300 mutations in bladder cancer remains unclear.

In this study, we focused on mutations in *EP300* and tested the functional consequences and underlying mechanisms of BCa-associated EP300 missense mutations for their transactivation activity and malignant transformation potentials.

## Results

### High throughput sequencing of bladder cancer reveals non-synonymous gene lesions

**Fig. 1:**
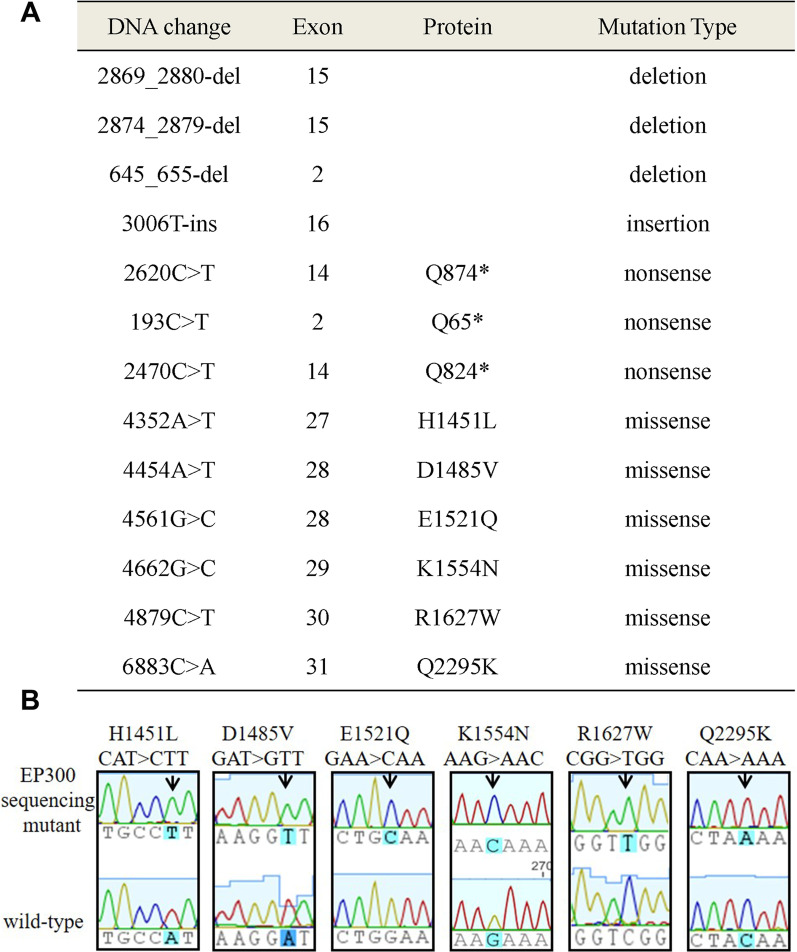
High-throughput sequencing of 97 cases with TCC. **A** High-throughput sequencing of bladder cancer reveals 13 non-synonymous gene lesions in EP300. **B** Sequencing traces of representative mutations and corresponding wild-type EP300 constructs. Arrows point to the position of the nucleotide change. Amino acid changes are shown at the top

We previously conducted high-throughput sequencing of 97 cases with TCC, which led to the identification of BCa-associated mutations in chromatin remodeling genes (Gui et al. 2011). We then focused on the typical chromatin remodeling gene, *EP300*. In total, 13 non-synonymous gene lesions in *EP300* were found, including 4 fragmental deletions/insertions, 3 nonsense mutations (mutations that result in premature stop codons), and 6 missense mutations (mutations that result in different amino acids) (Fig. 1A). Although fragment deletions/insertions and nonsense mutations are predicted to result in a complete inactivation or a loss of key functional domains in the final protein products, missense mutations that merely change a single amino acid in proteins might result in an indeterminate consequence. Therefore, six missense mutations, EP300-H1451L, D1485V, E1521Q, K1554N, R1627W, and Q2295K, were analyzed in this study (Fig. 1B).

**Fig. 2:**
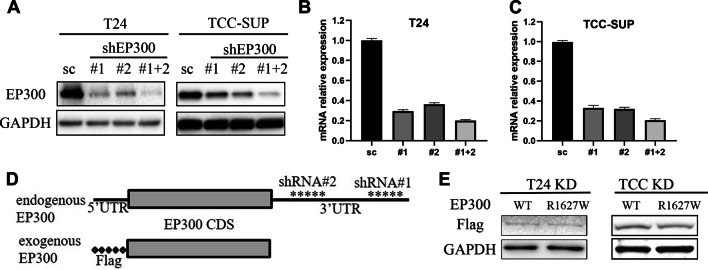
Establishment of EP300 knockdown bladder cancer cell lines.** A** EP300 protein level in T24 and TCC-SUP cell lines after transfection of EP300 shRNAs. **B, C** EP300 mRNA expression level after knockdown of EP300 in T24 and TCC-SUP. **D** Diagrams of EP300 knockdown strategy. Two shRNAs target endogenous EP300 3’UTR without targeting exogenous EP300 constructs. **E** Exogenous EP300-wt and EP300-R1627W are efficiently and equally expressed in T24-EP300kd cells

To investigate the functional consequences of these EP300 mutations, the corresponding FLAG-tagged expression plasmids, including EP300-wt, H1451L, D1485V, E1521Q, K1554N, R1627W and Q2295K were generated. Because endogenous EP300 proteins were reported to compromise the readout of exogenously expressed EP300-wt/mutations (Suganuma et al. 2002), we knocked down EP300 using two distinct lentiviral shRNAs against the 3’UTR region of EP300 mRNA (Fig. 2D). This approach enabled the efficient knockdown of endogenous EP300 at the mRNA and protein levels (Fig. 2A, B). Using this strategy, we established EP300-knockdown stable cell lines (T24-EP300kd and TCC-SUP-EP300kd) based on a T24 BCa cell line (T24-EP300wt) and a TCC-SUP BCa cell line (TCC-SUP-EP300wt), respectively, which served to minimize the influence of endogenous EP300 without interfering with the expression of exogenously reintroduced EP300 variants. Notably, exogenously reintroduced EP300-wt and EP300-R1627W were expressed at equal levels in T24-EP300kd cells and TCC-SUP-EP300kd cells (Fig. 2E).

### EP300-R1627W mutation impairs EP300 transactivation activity on both the p21 and p16 promoters

**Fig. 3:**
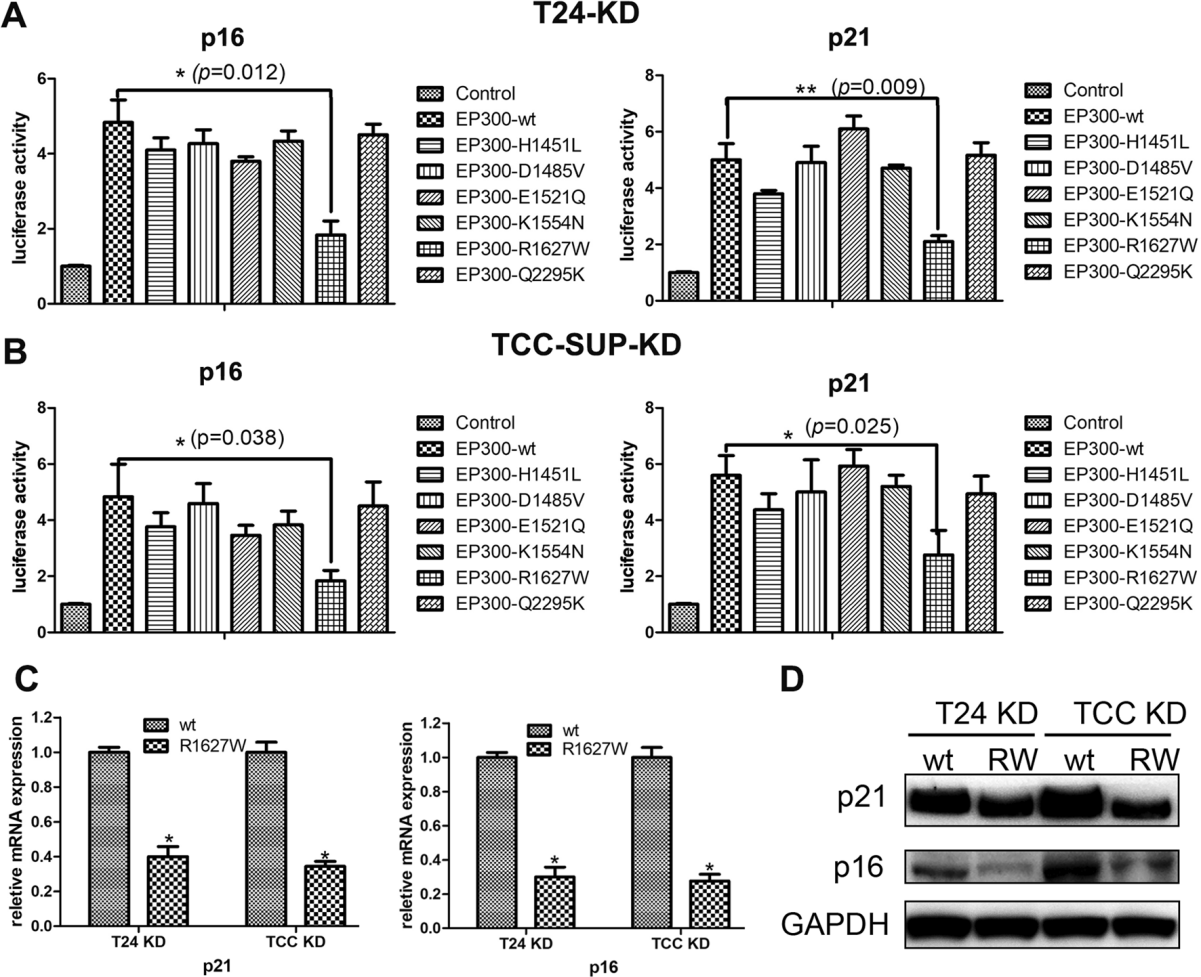
EP300-R1627W mutation impairs EP300 transactivation activity on both the p21 and p16 promoters. **A**, **B** Luciferase assays show that EP300-R1627W mutation impairs EP300 transactivation activity on both the p21 and p16 promoters in T24 and TCC-SUP. The bar graph shows results as relative activity compared to the control activity. **C**, **D** mRNA and protein expression of p21 and p16 after transfection of EP300-wt and EP300-R1627W plasmids in T24-EP300kd cells and TCC-SUP-EP300kd cells, respectively

Next, we assessed the transactivation activity of EP300-wt/mutants in T24-EP300kd cells and TCC-SUP EP300kd cells on the p21 and p16 promoters, which were both reported to be transcriptionally regulated by EP300 (Wang et al. 2008; Wong et al. 2010; Xiao et al. 2000). The results showed that EP300-wt, H1451L, K1554N, D1485V, and E1521Q increased the transcriptional activity of the p21 and p16 promoters. However, EP300-R1627W failed to activate the p21 and p16 promoters (Fig. 3A, B). Further experiments revealed that EP300-R1627W inhibited p16 and p21 mRNA and protein expression (Fig. 3C, D). In addition, to examine whether the effect of EP300-R1627W mutation followed an “all or none” pattern or “gradation” pattern, EP300-wt and EP300-R1627W plasmids were co-transfected into T24-EP300kd cells in different concentrations. The results showed that with the concentration of EP300-R1627W plasmids increased, the expression of p16 and p21 gradually decreased (Additional file 1: Fig. S1A, B). p21 promoter luciferase assay also showed that p21 transactivation activity decreased gradually when transfected with EP300-wt and EP300-R1627W plasmids (Additional file 1: Fig. S1C). These results demonstrated that the EP300-R1627W mutation in bladder cancer impaired EP300 transactivation activity following a “gradation” pattern.

### EP300-R1627W promotes BCa cell growth and invasion

**Fig. 4:**
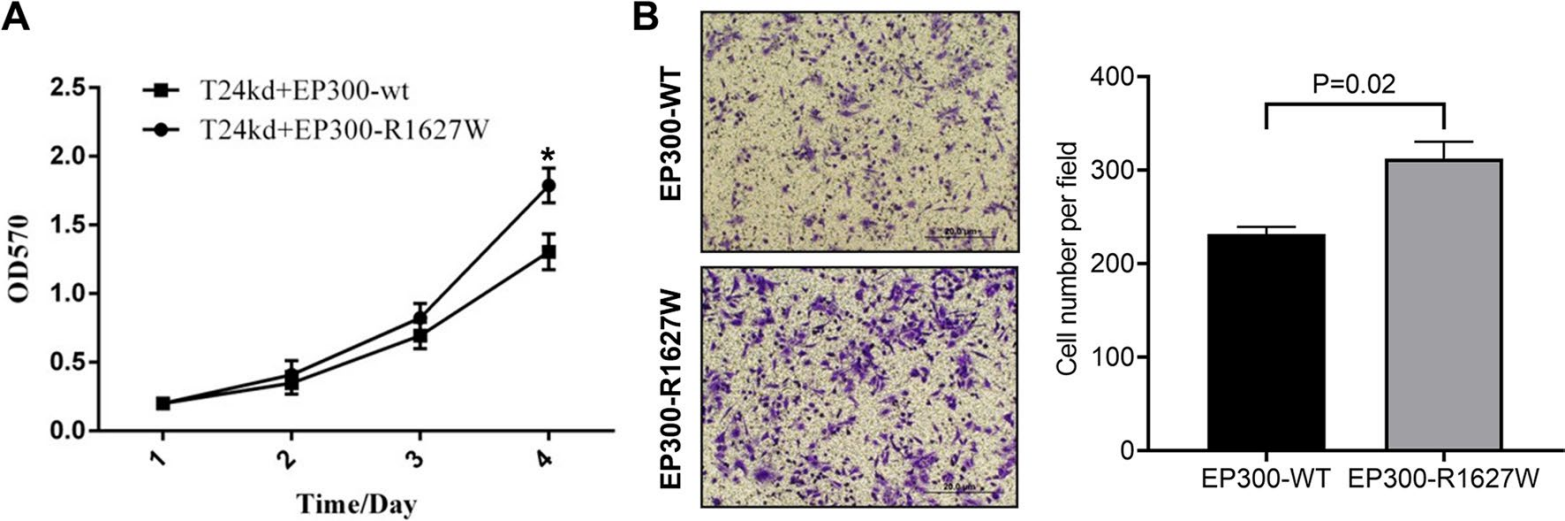
EP300-R1627W promotes BCa cell growth and invasion.** A** Growth curve of EP300-R1627W and EP300-wt in T24-EP300kd, respectively. **B** The invasion assay shows that cells containing EP300-R1627W are more aggressive than cells containing EP300-wt. Values are represented as mean ± SEM

In its function as a cofactor, EP300 can regulate the interaction between different proteins, therefore enabling it to regulate the expression of more than 16,000 genes in almost all cell types. As a result, EP300 can regulate proliferation, cell cycle, DNA damage response, and other cellular processes (Goodman and Smolik 2000; Hou et al. 2018). We then asked whether the EP300-R1627W mutation contributed to the malignant transformation of BCa. We examined the impact of EP300-wt/R1627W on BCa cell proliferation and invasion. EP300-wt and EP300-R1627W were reintroduced into T24-EP300kd cells using lentivirus infection. Stable cell lines expressing the two variants were selected for subsequent studies. In the proliferation assay, T24-EP300kd cells expressing EP300-R1627W grew significantly faster than cells expressing EP-300wt (Fig. 4A). In the invasion assay, as shown in Fig. 4B, expressing EP300-R1627W increased the invasion capacity of T24-EP300kd cells compared to EP300-wt. In addition, since EP300 is reported to have effects on the cell cycle, Flow cytometric analyses of cell cycle were performed to assess the effect of EP300-R1627W mutation on cell cycle. Compared to cells expressing EP300-wt, the proportion of S-phase and G2-phase cells was higher in cells expressing EP300-R1627W, indicating that EP300-R1627W cells were proliferating actively (Additional file 1: Fig. S2). These results demonstrated that EP300-R1627W contributed to the malignant growth invasion and cell cycle arrest of BCa cells, providing direct experimental evidence for the role of EP300-R1627W in BCa development and progression.

### R1627W mutation impairs EP300 HAT activity

**Fig. 5:**
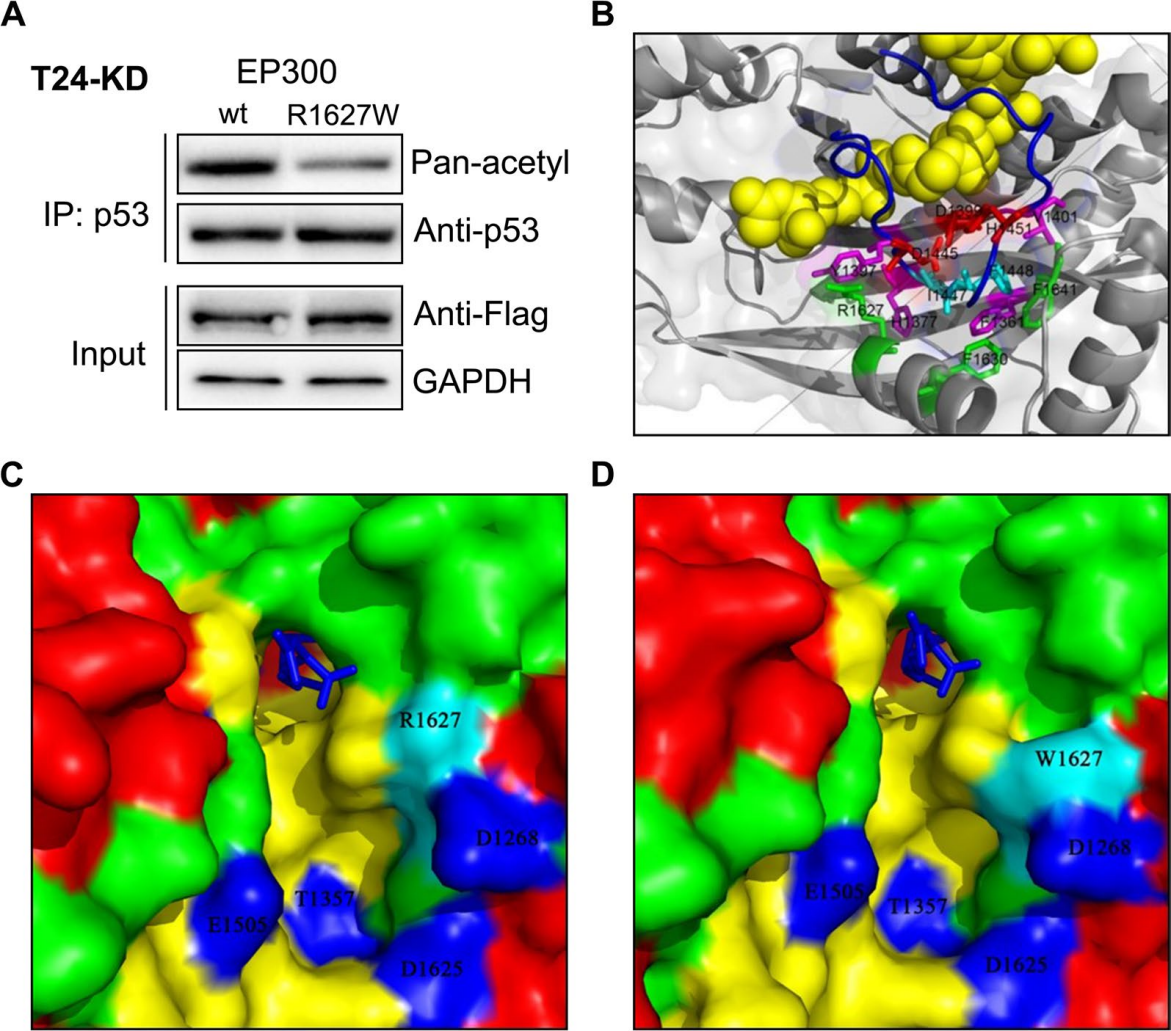
R1627W mutation impairs EP300 HAT activity by disrupting substrate-binding capacity. **A** Lower level of TP53 acetylation are found in cells expressing EP300-R1627W than those expressing EP300-wt. TP53 was first immunoprecipitated, and then TP53 acetylation was assessed using pan-lysine acetylation antibody. **B** R1627 positions in close to, but not direct contact with, the HAT catalytic core of EP300. A long loop (blue), together with its hydrogen-bonding interaction (red sticks) and hydrophobic interaction (pink sticks) amino acids, forms the catalytic core that embraces the substrate (yellow sphere). **C**, **D** Protein surface views of EP300-wt (left) and EP300-R1627W (right) catalytic core, which were analyzed and rendered using Pymol software. The side chain of T1357, E1505, D1625 and D1627 (blue) form a negative potential of a pocket for polypeptide substrate binding. R1627/W1627 is intimate facing this pocket. Side chain resulted from R > W substitution could greatly disrupt the electrostatic property of the pocket

EP300 comprises several domains. Mutations in different domains result in different consequences (Delvecchio et al. 2013). Sequence analysis revealed that R1627 is within the HAT domain of EP300. Therefore, we wanted to determine whether R1627W impaired the HAT activity of EP300. TP53 is a well-known tumor suppressor that was reported to be acetylated by EP300 (Gu and Roeder 1997), making it an ideal target for the analysis of EP300 acetyltransferase activity. We expressed EP300-wt or EP300-R1627W in the T24-EP300kd cell lines. Consequently, TP53 was immunoprecipitated and its acetylation status was determined. EP300-wt and EP300-R1627W were expressed at equal levels in T24-EP300kd cells, but acetylated TP53 was significantly decreased in cells expressing EP300-R1627W, compared to cells expressing EP300-wt (Fig. 5A). In addition, we also examined pan acetylation levels between cells expressing EP300-wt or EP300-R1627W. The results showed that EP300-R1627W mutation decreased pan acetylation levels (Additional file 1: Fig. S3). Taken together, these results indicated that EP300-R1627W leads to inefficient HAT activity.

Next, we investigated how R1627W, a single amino acid substitution, could impair the HAT activity of EP300. Pioneer works on EP300 structural dissection (Protein Data Bank ID: 4BHW (Delvecchio et al. 2013) and 3BIY (Liu et al. 2008)) offered us a precise 3D view of the EP300 HAT domain. As shown in Fig. 5B, the long loop (blue), together with its hydrogen-bonding interaction (red sticks) and hydrophobic interaction (pink sticks) amino acids, formed a catalytic core that embraces lysine-CoA (yellow spheres) (Liu et al. 2008). Mutations in the catalytic core greatly affect the enzymatic activity of EP300. For instance, EP300-D1399Y (red sticks) is an enzymatically dead variant (Ito et al. 2001). R1627 (green sticks) was spatially far from the amino acids that form the catalytic core, suggesting that the R1627W variant is less likely to lead to complete loss of HAT activity.

From the surface view of the catalytic core (Fig. 5C), the side chains of T1357, E1505, D1625, and D1628 (blue) form a negative potential of a pocket that is critical for polypeptide substrate binding. Structural modeling of EP300-R1627W was conducted based on the EP300-wt structure (3BIY) using the Swiss-model server (Kiefer et al. 2009). R1627 was positioned facing and adjoining the substrate-binding pocket (Fig. 5D). Notably, the R > W substitution—i.e., a polar basic amino acid arginine substituted with a nonpolar hydrophobic amino acid tryptophan—could greatly disrupt the affinity of this pocket to the polypeptide substrate. Taken together, the above analysis demonstrated that the R1627W mutation partially impaired EP300 HAT activity, potentially by interfering with its substrate-binding capacity.

### EP300-R1627W mutation promotes the growth of bladder cancer in vivo

**Fig. 6:**
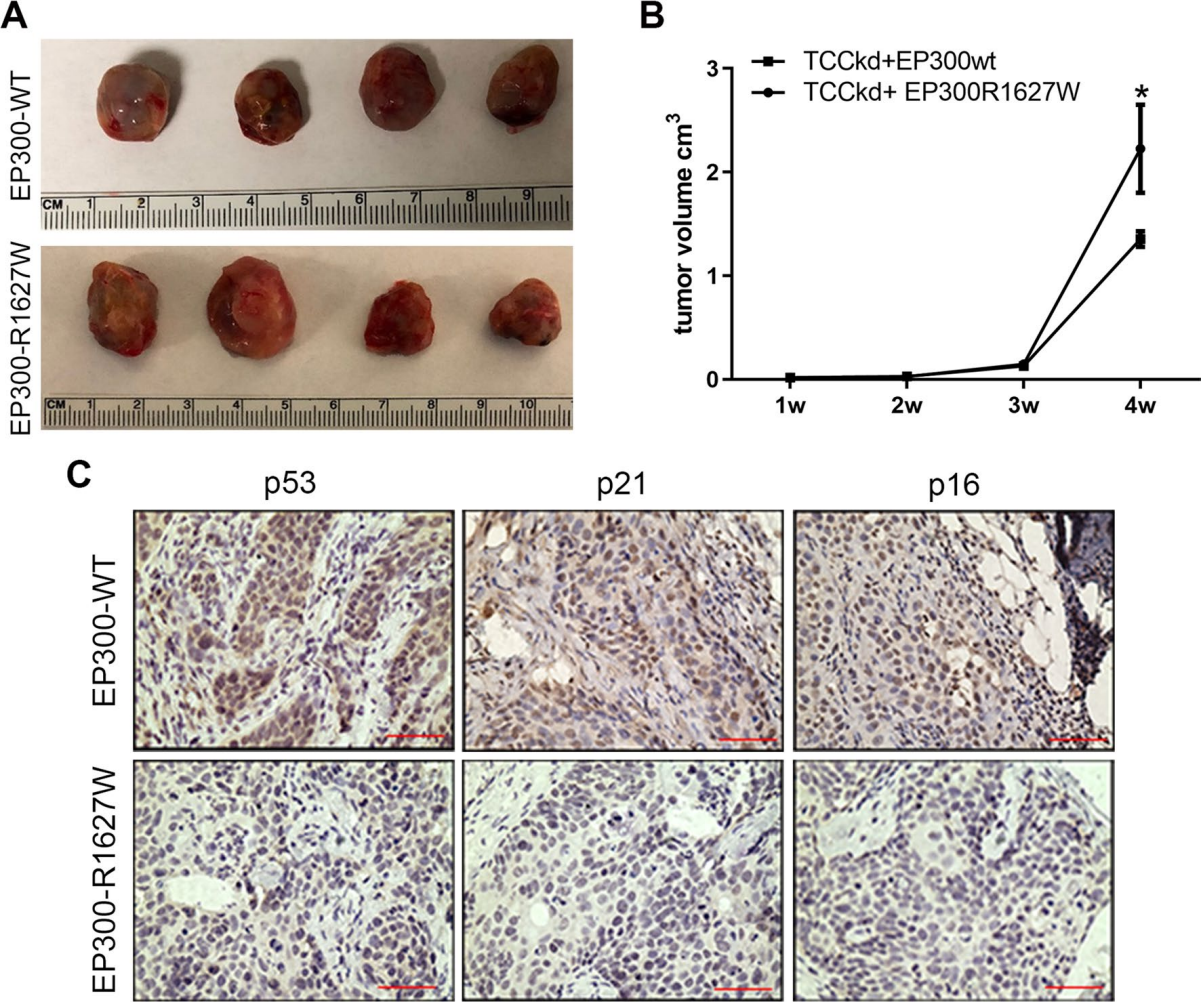
EP300-R1627W mutation promotes the growth of bladder cancer in vivo.** A** Images showing the size of the tumors that developed in mice of two groups. **B** Tumor volumes of two groups for 4 weeks after injection of EP300-wt-transfected and EP300-R1627W-transfected TCC-SUP-EP300kd cells, respectively. **C** Immunohistochemical analysis of p53, p16 and p21 expression level in harvested xenograft

To test the in vivo effects of the EP300-R1627W mutation in bladder cancer, TCC-SUP-EP300kd cells and TCC-SUP-EP300wt cells were grown as xenografts in nude mice. As a result, the EP300-R1627W mutation significantly promoted the growth of xenografts (Fig. 6A, B). Immunohistochemical analysis of harvested xenografts showed that p53, p16, and p21 expression was significantly decreased in EP300-R1627W xenografts (Fig. 6C). These results are consistent with our in vitro experiment, suggesting that the EP300-R1627W mutation might play a critical role in bladder cancer tumorigenesis.

### EP300-R1627W is recurrent in seven types of cancers

To determine the clinical relevance of EP300-R1627W, we performed data mining in the COSMIC (Catalog of Somatic Mutations in Cancers) database, based on the rationale that cancerous driver mutations are selected in cancer evolution and usually recurrent. Indeed, we found that the EP300-R1627W mutation was not only present in BCa, but also recurrent in seven other types of cancer: B-cell lymphoma, T-cell lymphoma, colon adenocarcinoma, endometrial carcinoma, serous carcinoma, malignant melanoma, and gastric adenocarcinoma (Additional file 2: Table S1). Notably, among the six BCa-associated EP300 missense mutants, EP300-R1627W was the only one that was recurrent in different cancers. Furthermore, R1627W was the second most frequent missense mutation among all detected mutations (data not shown). These findings suggested that EP300-R1627W might be a major player in tumorigenesis.

## Discussion

The current study expanded on our initial identification of several BCa-associated *EP300* mutations. Among these mutations, EP300-R1627W in particular was found to be ineffective in the transactivation of p21 and p16 promoters and inefficient in its ability to acetylate TP53 by interfering with the substrate-binding capacity of EP300. These functional changes are capable of promoting BCa growth and invasion. These results, combined with the knowledge that this mutation has been recurrent in seven different types of cancers, led us to conclude that EP300-R1627W is a driver mutation in BCa.

Large-scale genomic sequencing studies in cancer usually provide extensive lists of sequence variants, among which only a handful of gene mutations have a direct role in cancer (Prickett et al. 2014). With the development of modern sequencing technologies, large amounts of cancer-associated genetic lesions have been discovered, necessitating more functional efforts to translate the genomic information into mechanistic knowledge and clinical applications (Baudot et al. 2009). In this study, we provided direct experimental evidence to demonstrate that, among the six candidate missense mutations, EP300-R1627W acts as a driver of BCa progression. Consistent with our results, Duex et al., also found that EP300-R1627W was inefficient in acetylating histone H3 at lysine 27 in bladder cell lines, and structural analysis of EP300 also revealed that any substitution of R1627 residues would impair EP300 substrate binding capacity (Duex et al. 2018). Moreover, EP300-R1627W was predicted to have a strong impact on EP300 function using a well-known functional predictor of missense mutations, Mutation Assessor software (Betts et al. 2015). These findings provide robust evidence that mutations in chromatin-modifying enzymes contribute to tumorigenesis, as suggested in other studies (Le Gallo et al. 2012).

Interestingly, data mining in the COSMIC database revealed that EP300-R1627W was found not only in TCC BCa in our study, but also in B-cell lymphoma, T-cell lymphoma-leukemia, colon adenocarcinoma, endometrial carcinoma, serous carcinoma, malignant melanoma, and gastric adenocarcinoma. This finding underscored the driver role of the EP300-R1627W mutation. However, the driver role of EP300-R1627W in other cancers requires more evidence and studies.

Given the global involvement of EP300 in gene transcriptional regulation and multiple pathways, we cannot tell, based on current evidence, which cellular targets/pathways may be critically affected by EP300-R1627W. Because EP300-R1627W was inefficient in transactivating the p21 and p16 promoters, and acetylation of TP53 was decreased in cells expressing EP300-R1627W, we speculate that EP300-R1627W might disrupt TP53 pathways and cell cycle regulation pathways. Future work remains to fully understand the underlying mechanism of the EP300-R1627W mutation. Prospectively, we still need to clarify which pathway acts as the key pathway that EP300-R1627W mutation implicates in by RNA sequences. In addition, EP300 is a multifunctional protein whose functions are not limited to HAT activity. It is also possible that some EP300 mutations go through HAT activity-independent pathways to impact malignant transformation. Therefore, this study cannot exclude the possibility that other mutations on the list could also be driver mutations.

## Conclusion

In summary, our results demonstrated that among the BCa-associated EP300 missense mutations, EP300-R1627W acted as a driver. These results would have therapeutic implications in the view of current attempts to use histone acetylase/deacetylase inhibitors to treat BCa (Tanji et al. 2011; Verza et al. 2020).

## Materials and methods

### Oligos and vectors

All primers and oligos used in this study were synthesized by BGI Tech. (Shenzhen, China) and are listed in Additional file 2: Table S2. All clones were verified by Sanger sequencing.

FLAG-tagged wild-type EP300 cDNA was generated by PCR from a pCMV-HA-P300-wt plasmid (Song et al. 2012) and inserted into the EcoRI/BamHI site of pcDNA3.1 (pcDNA-FLAG-EP300-wt) using the In-Fusion® PCR Cloning System (Clontech, Mountain View, CA, USA). EP300 mutants were constructed from pcDNA-EP300-wt by the PCR-based site-direct mutation method, as described previously (Du et al. 2011). For lentiviral plasmids, FLAG-EP300-wt and the corresponding mutants were amplified by PCR and inserted into the pWPI-wt plasmid with XhoI/PmeI sites. To generate shRNA targeting EP300, two target sequences were selected from a previous study (Huang et al. 2009) and RNAi consortium (Root et al. 2006), respectively. Oligos were synthesized and inserted into the pLKO0.1 vector.

The human p21 promoter fragment (approximately 2700 bp) and human p16 promoter fragment (approximately 2900 bp) were amplified by PCR using primers as described in the Mammalian Promoter/Enhancer Database (accession ID RDB05893 and RDB05509). The PCR products were then inserted into the pGL3-basic vector using the NdeI/HindIII and MluI/XhoI sites, respectively.

### Cell lines, transfection, and luciferase assay

The BCa cell line T24 and TCC-SUP were purchased from the Type Culture Collection of the Chinese Academy of Sciences (Shanghai, China). Cells were grown in Dulbecco’s modified Eagle’s medium (DMEM) supplemented with 10% fetal bovine serum (ExCell Bio, China), 1% penicillin, and streptomycin. Plasmid transfection was performed using Lipofectamine 2000 (Invitrogen, Carlsbad, CA, USA). The Dual-Luciferase® Reporter Assay System (Promega, Madison, WI, USA) was used for the luciferase assay with pRL-TK as a control.

### Lentivirus and stable cell lines

Lentivirus was prepared as previously described (Cheng et al. 2012). Briefly, pLKO0.1 or pWPI, together with psPAX2 and pMD2.G packing vectors, were transfected into 293 T cells. The virus-containing medium was collected and centrifuged at 48 h after transfection. The resulting virus pellet was resuspended in PBS and used for infection of T24 cells in 6-well dishes in the presence of 5 µg/mL polybrene. Forty-eight hours after infection, the cells were ready for subsequent experiments. Cells expressing shEP300 were screened using puromycin at 2 µg/mL. Cells expressing EP300-wt/mutations were selected by observing the GFP fluorescence.

### Real-time qPCR

Cells were lysed by TRIzol Reagent (Invitrogen) to obtain RNA according to the manufacturer’s protocol. Reverse transcription was performed with 1 µg RNA using HiScript II Q RT SuperMix (Vazyme Biotech Co., Ltd). qPCR was performed using Taq Pro Universal SYBR qPCR Master Mix (Vazyme Biotech Co., Ltd) on QuantStudio 6 Flex system (Thermo Fisher Scientific). Data were analyzed using a 2–ΔΔCt method.

### Cell proliferation assay and invasion assay

Cells were seeded at a concentration of 5,000 cells per well in a 24-well plate and stained with 0.5% crystal violet at the indicated time points. Viable cells were quantified by measuring the absorbance at 570 nm. For the invasion assay, transwell chambers, equipped with a 6.5 mm diameter polycarbonate filter insert (pore size 8 µM) (Corning, NY, USA), were pre-coated with 200 µg/mL of Matrigel (BD Biosciences). The cells were seeded at a density of 105 cells/well. Twenty-four hours after seeding, cells that invaded through the Matrigel were fixed, stained, and quantified as previously described.

### Immunoprecipitation and Western blot

Immunoprecipitation was conducted as previously described. EP300 antibody (sc-584) and GAPDH antibody (sc-365,062) were purchased from Santa Cruz Biotechnology (Santa Cruz, CA, USA). Monoclonal ANTI-FLAG® M2 antibody (F3165) was purchased from Sigma-Aldrich (St. Louis, MO, USA). TP53 antibody (#9282) and acetylated-lysine antibody (#9814) were purchased from Cell Signaling (Beverly, MA, USA).

### Structural analysis

EP300 protein structure files were obtained from the Protein Data Bank (http://www.rcsb.org/pdb/home/home.do). The structure of EP300-R1627W was modeled using the SWISS-MODEL online server (http://swissmodel.expasy.org/). Protein structures were analyzed using the Pymol software.

### Xenograft tumor model

Ten million TCC-SUP EP300kd cells transfected with EP300-wt or EP300-R1627W plasmid, which were mixed 1:1 with Matrigel, were injected subcutaneously into the flanks of BALB/c nude mice (4–6 weeks, 18–20 g). Tumors were measured with a caliper once a week, and tumor volumes were calculated using length × width 2 /2. After 4 weeks, the tumors were harvested for photograph and immunohistochemistry staining. Data are expressed as mean ± SEM. The animal study was reviewed and approved by the Ethics Committee of the Nanfang Hospital, Southern Medical University.

### Immunohistochemistry staining

For immunohistochemical (IHC) staining, paraffin-embedded xenograft tissue sections were used according to the manufacturer’s protocols. Briefly, xenograft sections were dewaxed and rehydrated, and then the sections were incubated to block the endogenous peroxidase and were heated by microwave. After blocking, the slides were incubated with each primary antibody at 4℃ overnight, followed by secondary antibody incubation at room temperature for 1 h. Sections were then stained with diaminobenzidine (DAB) and counterstained with hematoxylin.

### Statistical analysis

The paired t-test was used for luciferase assay and invasion assay data. One-way ANOVA was used for comparisons among multiple groups. Each experiment was performed at least three times and the data are presented as means ± SD. Statistical significance was set at P < 0.05. *p < 0.05, **p < 0.01, ***p < 0.001. Statistical calculations were performed using GraphPad Prism software.

## Supplementary Information

**Additional file 1. Fig. S1.** The effect of EP300-R1627W mutation follows “gradation” pattern. **A** mRNA expression of p16 and p21 after co-transfection of EP300-wt and EP300-R1627W plasmids in T24-EP300kd cells. **B** protein expression of p16 and p21 after co-transfection of EP300-wt and EP300-R1627W plasmids in T24-EP300kd cells. C Luciferase assays of p21 promoter after co-transfection of EP300-wt and EP300-R1627W plasmids in T24-EP300kd cells. **Fig. S2.** EP300-R1627 mutation induced cell cycle arrest in bladder cancer cells. Flow cytometric analysis of cell cycle changes in T24-EP300kd cells after transfection of EP300-wt or EP300-R1627W plasmids. **Fig. S3.** EP300-R1627 mutation induced cell cycle arrest in bladder cancer cells. Western blot analysis of pan acetylation levels in cells transfected with EP300-wt or EP300-R1627W plasmids. 

**Additional file 2. Table S1.** EP300-R1627W is recurrent in other seven types of cancers. EP300-R1627W mutation was also found in B-cell lymphoma, T-cell lymphoma-leukemia, colon adenocarcinoma, endometrial carcinoma, serous carcinoma, malignant melanoma, and gastric adenocarcinoma. **Table S2.** Primer sequence list. All primers and oligos used in this study are listed in this table

## Data Availability

The data that support the findings of this study are available from the corresponding author upon reasonable request.

## Abbreviations

BCa: Bladder cancer
TCC: Transitional cell carcinoma
HAT: Histone acetyltransferase
COSMIC: Catalog of Somatic Mutations in Cancers
