# Supplementary material for: Experimental analysis of bladder cancer-associated mutations in EP300 identifies EP300-R1627W as a driver mutation
Source: Mol Med. 2023 Jan 16;29:7. doi: 10.1186/s10020-023-00608-7 (PMC9843983; doi:10.1186/s10020-023-00608-7)

Supplementary Figure.1 The effect of EP300-R1627W mutation follows "gradation" pattern. **A** mRNA expression of p16 and p21 after co-transfection of EP300-wt and EP300-R1627W plasmids in T24-EP300kd cells. **B** protein expression of p16 and p21 after co-transfection of EP300-wt and EP300-R1627W plasmids in T24-EP300kd cells. **C** Luciferase assays of p21 promoter after co-transfection of EP300-wt and EP300-R1627W plasmids in T24-EP300kd cells.

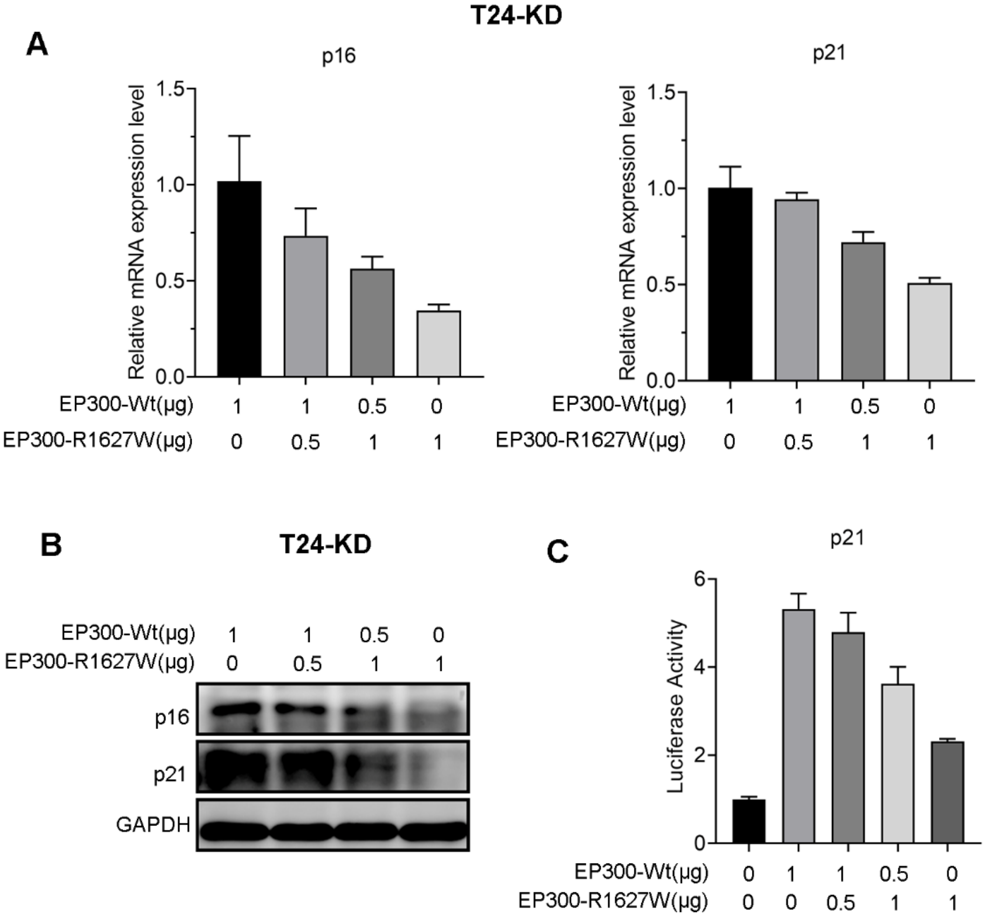

Supplementary Figure.2 EP300-R1627 mutation induced cell cycle arrest in bladder cancer cells. A Flow cytometric analysis of cell cycle changes in T24-EP300kd cells after transfection of EP300-wt or EP300-R1627W plasmids.

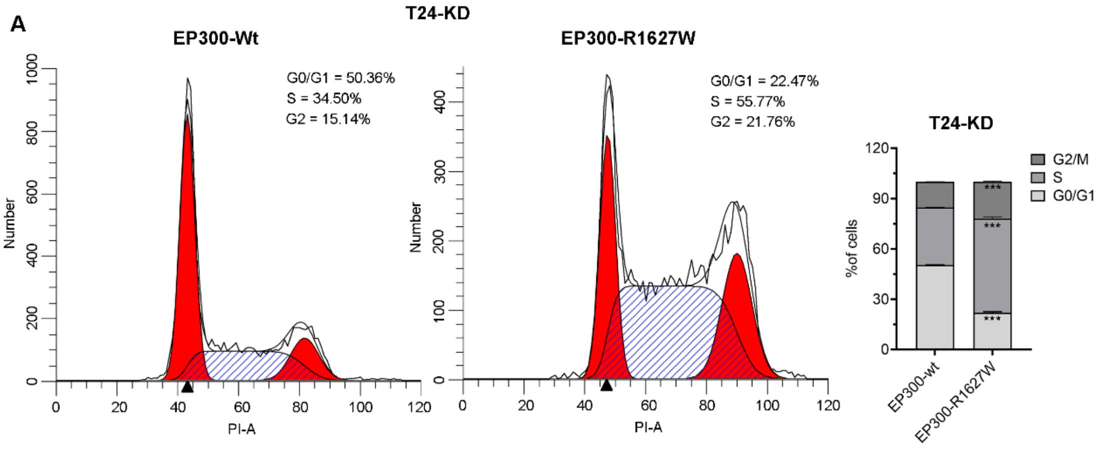

Supplementary Figure.3 EP300-R1627 mutation induced cell cycle arrest in bladder cancer cells. A Western blot analysis of pan acetylation levels in cells transfected with EP300-wt or EP300-R1627W plasmids.

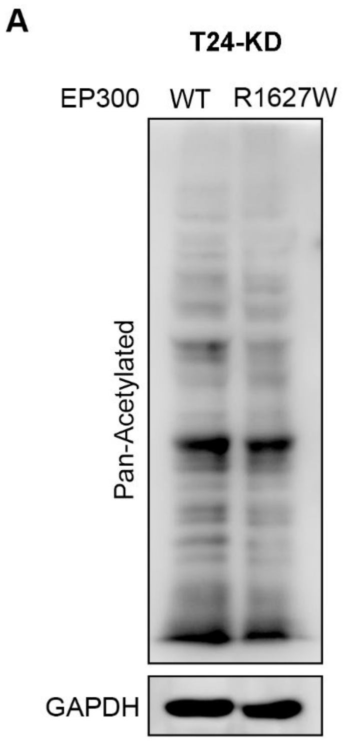

Supplement: Supplementary file 1 — Additional file 1. Fig. S1. The effect of EP300-R1627W mutation follows “gradation” pattern. A mRNA expression of p16 and p21 after co-transfection of EP300-wt and EP300-R1627W plasmids in T24-EP300kd cells. B protein expression of p16 and p21 after co-transfection of EP300-wt and EP300-R1627W plasmids in T24-EP300kd cells. C Luciferase assays of p21 promoter after co-transfection of EP300-wt and EP300-R1627W plasmids in T24-EP300kd cells. Fig. S2. EP300-R1627 mutation induced cell cycle arrest in bladder cancer cells. Flow cytometric analysis of cell cycle changes in T24-EP300kd cells after transfection of EP300-wt or EP300-R1627W plasmids. Fig. S3. EP300-R1627 mutation induced cell cycle arrest in bladder cancer cells. Western blot analysis of pan acetylation levels in cells transfected with EP300-wt or EP300-R1627W plasmids. [file 10020_2023_608_MOESM1_ESM.pdf]
