# Supplementary material for: Experimental analysis of bladder cancer-associated mutations in EP300 identifies EP300-R1627W as a driver mutation
Source: Mol Med. 2023 Jan 16;29:7. doi: 10.1186/s10020-023-00608-7 (PMC9843983; doi:10.1186/s10020-023-00608-7)
Supplement: Supplementary file 2 — Additional file 2. Table S1. EP300-R1627W is recurrent in other seven types of cancers. EP300-R1627W mutation was also found in B-cell lymphoma, T-cell lymphoma-leukemia, colon adenocarcinoma, endometrial carcinoma, serous carcinoma, malignant melanoma, and gastric adenocarcinoma. Table S2. Primer sequence list. All primers and oligos used in this study are listed in this table [file 10020_2023_608_MOESM2_ESM.pdf]

Supplementary Table.S1 EP300-R1627W is recurrent in other seven types of cancers. EP300-R1627W mutation was also found in B-cell lymphoma, T-cell lymphoma-leukemia, colon adenocarcinoma, endometrial carcinoma, serous carcinoma, malignant melanoma, and gastric adenocarcinoma.

Supplementary Table. S1

Gene name	Protein change	Primary tissue	Histology	Sample name
EP300	R1627W	Urinary tract	Transitional cell carcinoma, bladder cancer	B86
EP300	R1627W	Stomach	Adenocarcinoma	TCGA-BR-A4QL-01
EP300	R1627W	Skin	Malignant melanoma	TCGA-D3-A2JF-06
EP300	R1627W	Large intestine	Colon Adenocarcinoma	TCGA-AA-A010-01
EP300	R1627W	Endometrium	Endometrioid carcinoma	P-0011509-T01-IM5, TCGA-AX-A3FT-01
EP300	R1627W	Endometrium	Serous carcinoma	T3(OM1323)
EP300	R1627W	Lymphoid tissue	B-cell lymphoma	SUDHL6, 7_tFL
EP300	R1627W	Lymphoid tissue	T cell lymphoma-leukemia	ATL171

Table.S2 Primer sequence list. All primers and oligos used in this study are listed in this table.

Primers for EP300 cloning	5' to 3'
EP300-infusion-F	GATATCTGCAGAATTCATGGCCGAGAATGTGGTGGAAC
EP300-infusion-R	TACCGAGCTCGGATCCCTAGTGTATGTCTAGTGTACTCT
primers for EP300 mutation construction	5' to 3'
1451-F	ATATCTTCCATTGCCTTCCTCCTGACCAGAAGATAC
1451-R	TTCTGGTCAGGAGGAAGGCAATGGAAGATATAATCA
1485-F	TCCATGACTACAAGGTTATTTTTAAACAAGCTACTG
1485-R	TAGCTTGTTTAAAAATAACCTTGTAGTCATGGACAATAC
4519-F	CCTTATTTCGAGGGTGATTTCTGGCCCAATGTTCTG
4519-R	CATTGGGCCAGAAATCACCCTCGAAATAAGGCAATTC
1521-F	AGCATTAAGGAACTGCAACAGGAGGAAGAAGAGAGA
1521-R	CTTCTTCCTCCTGTTGCAGTTCCTTAATGCTTTCTT
1554-F	AAAAGAAGAATAATAACAAAACCAGCAAAAATAAGAG
1554-R	ATTTTTGCTGGTTTTGTTATTATTCTTCTTTTTAGCA
1627-F	GATCTGATGGATGGTTGGGATGCGTTTCTCACGCTG
1627-R	GATCTGATGGATGGTTGGGATGCGTTTCTCACGCTG
2295-F	CAGTCCCCACACCTAAAAGGCCAGCAGATCCCTAA
2295-R	GATCTGCTGGCCTTTTAGGTGTGGGGACTGGGCCTGA
1399-F	CATATCTTACCTCTATAGTGTTCATTTCTTCCGTC
1399-R	GAAATGAACACTATAGAGGTAAGATATGTATACTCTC
primers for quantitative PCR	5' to 3'
qEP300-F	AGCCAAGCGGCCTAAACTC
qEP300-R	TCACCACCATTGGTTAGTCCC
GAPDH-F	CTCCTCACAGTTGCCATGTA
GAPDH-R	GTTGAGCACAGGGTACTTTATTG
primers for promoter cloning	5' to 3'
NdeI-p21-F	CGCCATATGAAAAGCCAGGGCTGCCTCTGCTCAA
Hind3-p21-R	GCGAAGCTTATCCGCGCCCAGCTCCGGCTCCACA
MluI-p16-F	CGCACGCGTATCTCCCATCTTCTTCACCAATAGC
XhoI-p16-R	CGCCTCGAGGACCCTGTCCCTCAAATCCTCTG
primers for EP300 sequencing	5' to 3'
p300-580-F	AACGGTTCAATTGGAGCAGG
p300-640-R	TAAGTTGCCAGCACTTCCCA

p300-1380-F	AGTCAGATTGATCCCAGCTCC
p300-1900-R	CCGCTCGATTGTTTGCAGAT
p300-3800-F	TGGCCTGCTGGATTCGTCTG
p300-3900-R	AGAAAGGTGCCAAGTCTGGT
p300-4620-F	TGACCAAGGGAGACAGCAAA
p300-5300-F	GTGCAGCATACCAAGGGTTG
p300-6650-F	AACCCCAAGGAGTTGGCTAC
p300-6890-R	TTAGGGATCTGCTGGCCTTG
